# Characterization of a ViI-like Phage Specific to *Escherichia coli *O157:H7

**DOI:** 10.1186/1743-422X-8-430

**Published:** 2011-09-07

**Authors:** Elizabeth M Kutter, Kyobi Skutt-Kakaria, Bob Blasdel, Ayman El-Shibiny, Anna Castano, Daniel Bryan, Andrew M Kropinski, Andre Villegas, Hans-Wolfgang Ackermann, Ana L Toribio, Derek Pickard, Hany Anany, Todd Callaway, Andrew D Brabban

**Affiliations:** 1 The Evergreen State College, Olympia, WA, USA; 2 Faculty of Environmental Agricultural Sciences, Suez Canal University, Egypt; 3 Laboratory for Foodborne Zoonoses, Public Health Agency of Canada, Guelph, ON, Canada; 4 Department of Molecular & Cellular Biology, University of Guelph, ON, Canada; 5 Department of Microbiology, Faculty of Medicine, Laval University, Quebec, QC, Canada; 6 The Wellcome Trust Sanger Institute, Genome Campus, Hinxton, England, UK; 7 Canadian Research Institute for Food Safety, University of Guelph, ON, Canada; 8 Microbiology Department, Ain Shams University, Cairo, Egypt; 9 USDA Agricultural Station, College Station, TX, USA; 10 Department of Microbiology, The Ohio State University, Columbus, OH; 11 Department of Pediatric Neurology, University of Colorado Children's Hospital, Denver, CO

**Keywords:** *E. coli *O157:H7, hydroxymethyluracil, phage evolution, phage ecology, genome, proteome, bioinformatics, Vi antigen, O157 antigen, tail spike, T4 core genes

## Abstract

Phage vB_EcoM_CBA120 (CBA120), isolated against *Escherichia coli *O157:H7 from a cattle feedlot, is morphologically very similar to the classic phage ViI of *Salmonella enterica *serovar Typhi. Until recently, little was known genetically or physiologically about the ViI-like phages, and none targeting *E. coli *have been described in the literature. The genome of CBA120 has been fully sequenced and is highly similar to those of both ViI and the *Shigella *phage AG3. The core set of structural and replication-related proteins of CBA120 are homologous to those from T-even phages, but generally are more closely related to those from T4-like phages of *Vibrio, Aeromonas *and cyanobacteria than those of the *Enterobacteriaceae*. The baseplate and method of adhesion to the host are, however, very different from those of either T4 or the cyanophages. None of the outer baseplate proteins are conserved. Instead of T4's long and short tail fibers, CBA120, like ViI, encodes tail spikes related to those normally seen on podoviruses. The 158 kb genome, like that of T4, is circularly permuted and terminally redundant, but unlike T4 CBA120 does not substitute hmdCyt for cytosine in its DNA. However, in contrast to other coliphages, CBA120 and related coliphages we have isolated cannot incorporate ^3^H-thymidine (^3^H-dThd) into their DNA. Protein sequence comparisons cluster the putative "thymidylate synthase" of CBA120, ViI and AG3 much more closely with those of *Delftia *phage φW-14, *Bacillus subtilis *phage SPO1, and *Pseudomonas *phage YuA, all known to produce and incorporate hydroxymethyluracil (hmdUra).

## Background

In recent years, as concerns about antibiotic resistance and food safety have escalated, researchers have become interested in the use of bacteriophages to detect and combat bacterial contamination of food products [1-6]. Cocktails of obligately lytic phages have been applied to control such problematic foodborne pathogens as *Escherichia coli *O157:H7, *Shigella, Salmonella, Listeria *and *Campylobacter*, though with mixed success [7-16]. Unfortunately, most of the phages used to date in field trials have undergone little characterization. A better understanding of the properties of the individual phages and of their interactions with the host and each other during co-infection is important if phage-mediated biocontrol is to be successful.

A number of different phages targeting *E. coli *have been isolated and intensely studied but few if any of the well-characterized ones infect serotype O157:H7. We have previously described the isolation, characterization, distribution and inter-relationships between *E. coli *O157:H7 and phages isolated from sheep and cattle yards that efficiently infect this pathogen [12,13,17,18]. The cyclical nature of the *E. coli *O157:H7 levels seen in cattle and the inverse correlation between this bacterium and phage levels suggests that endogenous phages provide a natural control mechanism [12,16,18]. We have found only three types of phages among the many isolated against O157:H7 from sheep and from cattle feedlots: T4-like myoviruses such as CEV1 [13], myoviruses distinct from T4 in morphology and behavior, and T5-like siphoviruses such as CEV2 [12]. Here we present a detailed characterization of vB_EcoM_CBA120, a phage belonging to the second group.

## Results

### Isolation and Initial Characterization of the CBA Set of Phages Targeting *E. coli *O157:H7

The systematic isolation and quantitation of *E. coli *O157:H7 and a group of phages targeting this bacterium from stockyards in the southwest US was described by Callaway et al., [17] and by Oot et al., [18]. CBA120 is one of these phages. It is of particular interest since it is the first phage we have encountered which appears to be highly specific for *E. coli *O157:H7, infecting only one of the 72 ECOR collection strains -- ECOR 70 (078:H^-^) -- in addition to at least 13 out of 17 tested O157:H7 strains. In contrast, the T4-like and T5-like phages we have isolated from ruminant sources infect most of the tested O157 isolates, and each of those phages infects at least eight members of the ECOR collection [12,13]. They each also infect *E. coli *B and K12, which are not targeted by CBA120. CBA120 did not plate on any of 107 tested *E. coli *strains from cows with metritis, while T4-like CEV1 plated on 9 of them and T5-like CEV2 plated on 7, all but 1 different from each other (unpublished data).

### Morphology of Phage CBA120

**Figure 1:**
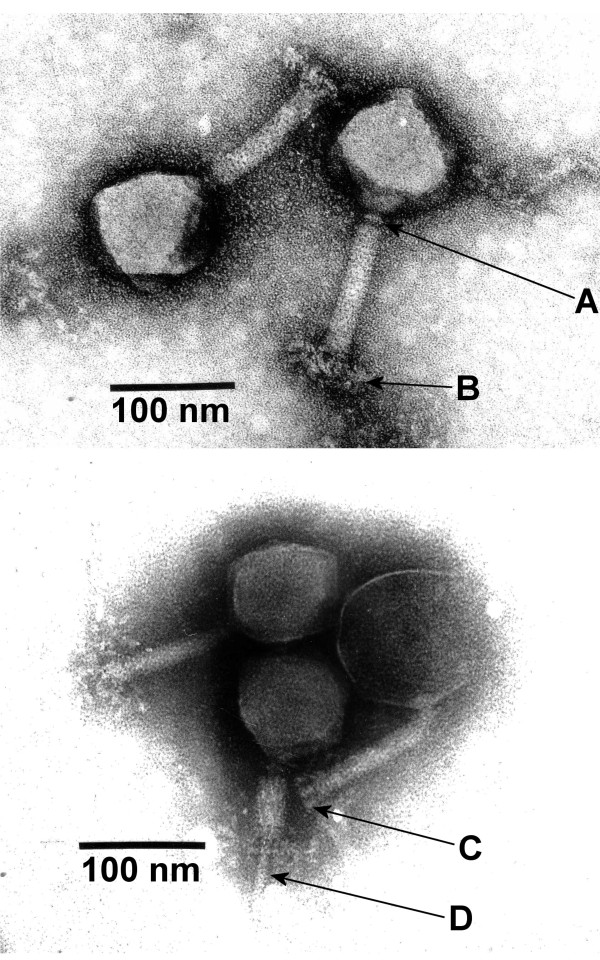
**Transmission electron micrographs of CBA120 particles stained with uranyl acetate (top) or phosphotungstic acid (bottom)**. Arrows indicate: A, neck with collar; B, reticular structure around the baseplate; C; tail spikes; D, tail tube after sheath contraction.

Phage CBA120 (Figure 1) has an isometric head ~90 nm in diameter and a contractile tail of ~105 × 17 nm, which measures ~42 × 23 nm in the contracted state (arrow D). Tails show transverse striations, a neck, a tiny collar (arrow A), a baseplate, and terminal spikes which may unfold into a complex system of fibers (arrows C, B). Its structure is very similar to that of *Salmonella *Typhi phage ViI [19,20].

### Aerobic and Anaerobic Physiological Studies

**Figure 2:**
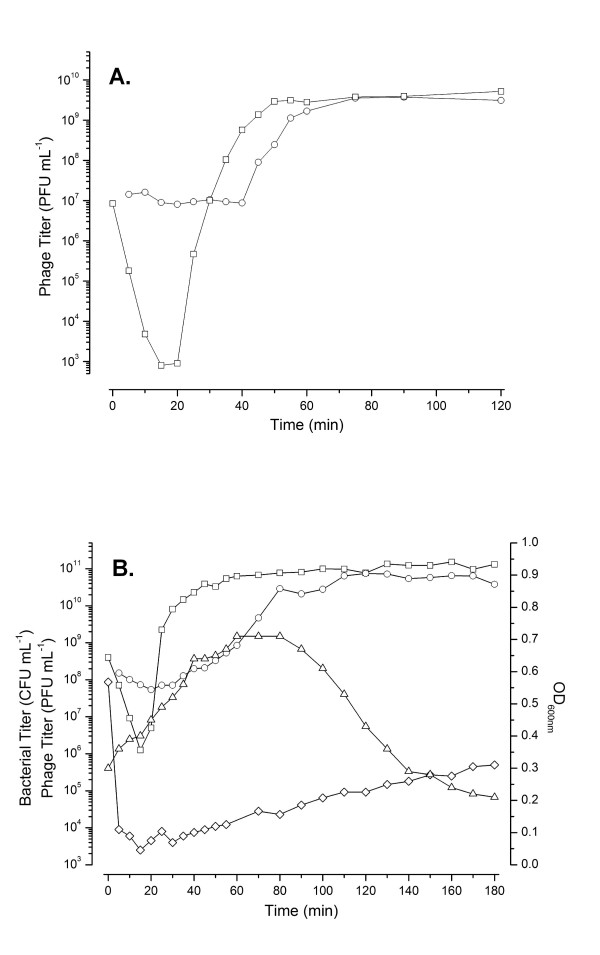
**Effect of multiplicity of infection of phage CBA120 on host cells**. 2A. Low MOI (~0.1) single-step growth curve of CBA120 infecting *E. coli *O157:H7 NCTC 12900. The infection graph shown is representative of three replicates. Infective centers (PFU mL^-1^, O), and total viable phage (PFU mL^-1^, □, as determined after the addition of chloroform). 2B. High MOI (~10) CBA120 infection of *E. coli *O157:H7 NCTC 12900. The infection graph shown is representative of three replicates. Infective centers (PFU mL^-1^, O), phage (PFU mL^-1^, □ after the addition of chloroform), optical density (600 nm, Δ) and bacterial titer/survivors (CFU mL^-1^, ◇).

**Figure 3:**
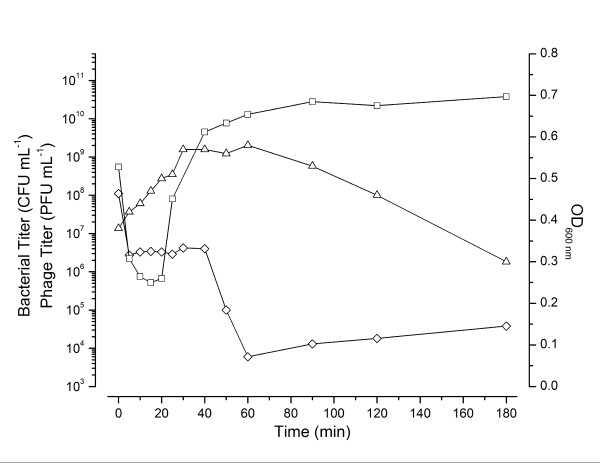
**Anaerobic (MOI ~3) CBA120 infection of *E. coli *O157:H7 NCTC 12900 exponentially growing in TSB at 37°C under an N_2 _headspace**. Symbols as in Figure 2.

CBA120 adsorbs rapidly to *E. coli *O157:H7 at 37°C. Aerobic single-step growth experiments, carried out at a low multiplicity of infection (MOI) (Figure 2A), indicate an eclipse period of ~20 min and a latent period of ~40 min. By 50 min, phage production appears to have largely ceased after generating an average burst of ~440 phage per cell (standard error = 18). All phage have been released by 80 min after infection. However, when the infection is carried out at high MOI, the turbidity (OD_600 nm_) of the culture continues to increase long after infection, almost tripling before gradual lysis is initiated at about 90 min, releasing 1000-1300 phage per cell (Figure 2B). Anaerobically, we observed the same relatively late gradual lysis beginning by 90 min (Figure 3), with an average of ~100 phage per cell present at 60 min and ~250 phage per cell at 250 min. However, we routinely observed that ~40 min after infection some phage are abruptly released, as seen by the rapid loss of the remaining susceptible bacteria, both anaerobically (Figure 3) and aerobically (data not shown) when there are sufficient survivors to observe this phenomenon (at MOIs of 2-5). This difference in lysis time and burst size between high and low MOI aerobic infections is reminiscent of the lysis inhibition phenomenon observed in T4.

### Tritium Labeling of Phage DNA

**Figure 4:**
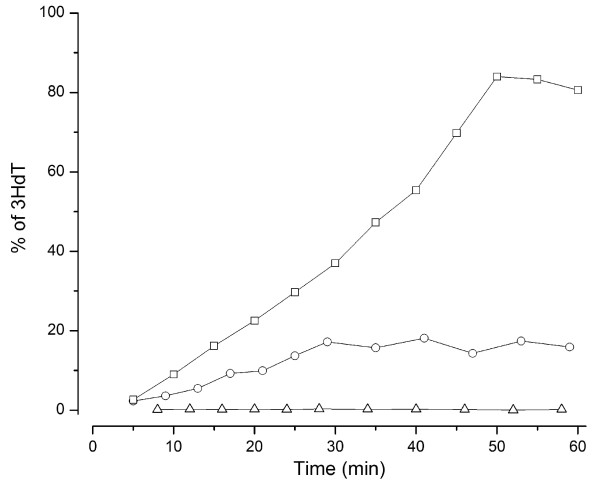
**Comparison of ^3^H-dThd uptake in uninfected *E. coli *12900 (O) and after infection with phage CBA120 (Δ) or CEV1 (□)**.

In extensive previous work we found that T5-like phage CEV2 blocks CBA120 phage production (unpublished data). To determine whether this blockage involved degradation of the CBA120 DNA in the co-infected host, we tried to make a stock of CBA120 labeled with tritiated thymidine (^3^H-dThd). Most phages that we have studied readily incorporate dThd into their DNA, paralleling phage production, so long as deoxyadenosine (dAdo) is added to block the cleavage of the thymidine's glycosidic bond [21]. In the experiment shown in Figure 4, a culture of exponentially-growing *E. coli *O157:H7 NCTC 12900 was infected with CBA120 or with T4-like phage CEV1. Five min post-infection, 2 mL of the infected culture was transferred to a 10 mL flask containing the ^3^H-dThd and dAdo. Surprisingly, no label was incorporated during infection with CBA120, although a large burst of phage was produced and isotope was incorporated as expected in the controls: an uninfected culture and one infected with CEV1. Efficient isotope uptake was also observed for the T5-like CEV2, while no incorporation was seen with the CBA120-like phage CBA6 (data not shown). The most likely explanation is that the CBA120 group of phages uses some base other than dThd, analogous to T4's substitution of hydroxymethylcytosine (hmdCyt) for cytosine.

### GENOMIC ANALYSIS OF CBA120

**Figure 5:**
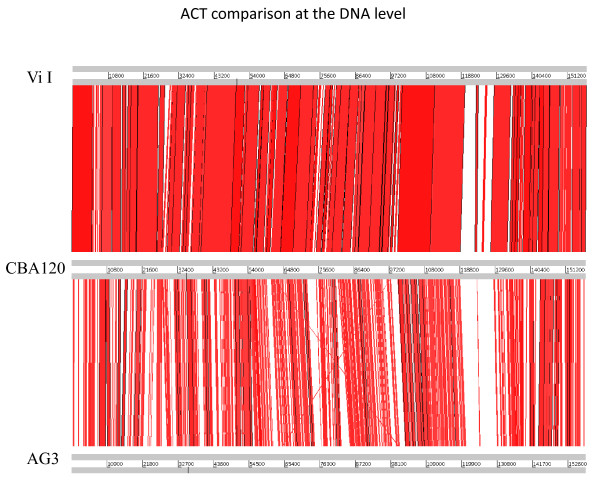
**DNA-level ACT alignment of the genomes of phages ViI (top), CBA120 and AG3**. Regions in red represent high DNA sequence similarity between the genomes.

The CBA120 genome was found to be 157,304 bp long and circularly permuted, with a GC content of 44.5%. It is very similar to the genomes of the classic *Salmonella enterica *serovar Typhi typing phage ViI [20] and *Shigella *phage AG3 [22] (Table 1). Whole-genome comparisons, made at both the DNA and the protein level using Artemis Comparison Tool (ACT) [23], show a remarkable degree of synteny between the three phages (Figure 5). As determined by reciprocal FASTA comparisons, 147 genes are conserved among all three phages, with only 17 being unique to the genome of CBA120. No homologs of virulence factor or lysogeny-associated genes have been found.

**Table 1:** Comparison of the genomic properties of phages CBA120, ViI and AG3

Phage	CBA120	ViI	AG3
Genome size (bp)	157,304	157,061	158,006
G+C (%)	44.5	45	50.4
Predicted genes	202	208	216

**Figure 6:**
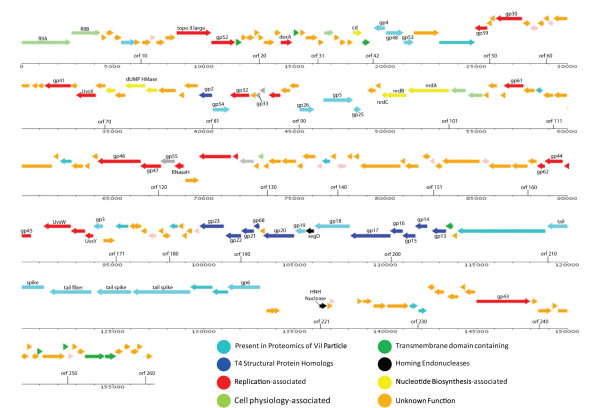
**Functional genomic map of CBA120**. The "gp" (gene product) numbers refer to the corresponding gene numbers of bacteriophage T4. Red: DNA replication; yellow: nucleotide metabolism; dark blue: head-associated; turquoise: tail-associated; medium blue: orthologs seen in ViI proteomic analysis, but otherwise unidentified; grey: regulatory genes.

The major difference between the three phages seen in the ACT depiction of Figure 5 lies, not surprisingly, in the region of the complex tail spikes and fibers (CBA120 orfs 210-213, as seen in Figure 6; genes Vi0I_170c-174c; and AG3 orfs 207, 210 and 212) -- the only region of ViI explored in detail by Pickard et al., 2010 [20] in their genomic analysis of the seven classic Vi typing phages which target the *Salmonella enterica *serovar Typhi Vi antigen. Though not orthologous, these four proteins are surprisingly similar in general structure between CBA120, ViI and AG3, while being totally different from those of any other group of myoviruses reported to date. Since most of the ViI genome has not yet been otherwise discussed, we include it with CBA120 in this detailed genomic analysis.

### Core Gene Analysis

Genomic analysis indicates that phages CBA120, ViI and AG3 carry all but one of the 39 genes which constitute the T4 superfamily core genome (Figure 6). These core genes, determined by comparing the genomes of coliphages T4, RB43, and RB49, *Aeromonas salmonicida *phage 44RR2.8t, and *Prochlorococcus *phage P-SSM2, are by convention given their T4 names, taking advantage of the extensive studies of T4 in determining their function [24-28]. Twenty one of these core genes encode proteins involved in capsid and tail formation with most of the rest related to nucleic acid metabolism and control of late transcription. The homologies extend well beyond these core genes; CBA120 shares 69 homologues with *Delftia acidovorans *phage ФW-14, 59 with *Aeromonas *phage Aeh1, 53 with T4, and 50 with cyanophage P-SSM2, but the gene order is very different in each of these phages.

### Transcription and Translation

Phages CBA120, ViI and AG3 all have homologs of gp55, 33 and 45, which are required for transcription of the T4 late genes [29], suggesting that the mechanism for controlling structural-gene transcription is similarly complex (see Discussion). The T4 late promoter consensus sequence is simply TATAAATA, while in the case of the T4-like cyanophages, this is NATATAATA [30]. The sequence recognized by the late transcription complex has not yet been identified for the ViI family of phages. Regulation of early- and middle-mode transcription in CBA120 is much less clear. Based on sequence similarity to the consensus σ^70 ^promoter, TTGACA (N_15-18_) TATAAT, only eight probable promoters were identified. The genomic layout makes it clear that there must be many additional promoters functioning early in infection to direct the transition from host to viral metabolism. At the translation/RNA level, only orthologs of RNase H have been identified, but this is the level of regulation least well understood in the T4 family [27]. CBA120 also encodes tRNAs for Met: (CAU), Asn (GUU), Ile (GAU) and Ser (GCU), followed by what appears to be a degenerate tRNA of undetermined specificity. ViI and AG3 have similarly located clusters of a few tRNAs.

### Homing Endonucleases

CBA120 has only two putative homing endonucleases, encoded by orf 195 (a homolog of T4 segD) and orf 221. The latter is a shortened helix-turn-helix (HN-H) endonuclease, which overlaps the predicted Met tRNA and is adjacent to an unusual 2 kb region where no genes have been identified (Figure 6). Similarly, phages ViI and AG3 have only 2 or 3 such homing endonucleases.

### DNA Replication and Nucleotide Metabolism

Most nucleotide metabolism genes are homologous to the corresponding genes in the T-even phages: the B-family DNA polymerase and its accessory proteins: a single-stranded DNA binding protein, sliding clamp and clamp loader, helicases, primase, exonuclease, and DNA ligase; the A, B and C subunits of ribonucleotide reductase; dCMP deaminase; and dUTPase/dUDPase.

**Figure 7:**
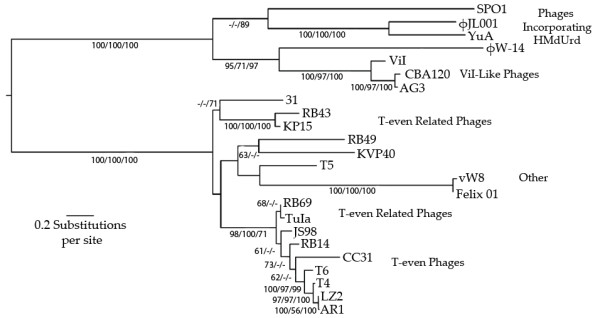
**Phylogenetic tree of genes initially identified as "Thymidylate Synthase" (TS) or "Thymidylate Synthetase" by homology searches of the non-redundant NCBI protein database**. The topology and branch lengths correspond to the Maximum Likelihood (ML) inference. Bootstraps appear ordered as ML, Neighbor Joining and Maximum Parsimony. Only bootstrap values greater than 50 are shown. All of the phages are members of the *Myoviridae *except for siphoviruses T5, infecting *E*. coli, YuA, infecting *Pseudomonas*, and JL001, targeting a marine γ-Proteobacterium. Phage SPO1 infects *B. subtilis*, Felix O1 infects many species of *Salmonella *and wV8 infects *E. coli*. The rest of the phages belong to the T4 superfamily, most of them infecting *E. coli *and some strains of *Shigella*. However, KVP40 infects *Vibrio parahaemolyticus*, 31 infects *A. salmonicida*, φW-14 infects *D. acidovorans*, and KP15 infects *Klebsiella pneumoniae*. Coliphage LZ2 was isolated by Sean Eddy from the Denver zoo and several segments were sequenced in the Kutter lab, in comparison with those from a number of other T4-like phages [44].

In addition, we found a putative thymidylate synthase (TS; orf 073), adjacent to which is an apparent dNMP kinase (orf 070). In light of the above-noted failure of CBA120 to incorporate ^3^H-dThd into its DNA, the putative TS of CBA120 was carefully examined. BLAST analysis against the non-redundant protein databases shows that this protein is far more similar to the deoxyuridylate hydroxymethyltransferase (dUMP HMase) seen in phages that have been shown to contain hmdUra in their DNA rather than dThd (Figure 7). This includes proteins of *Bacillus subtilis *phage SPO1 and *Delftia acidovorans *phage φW-14 [31,32]. In the latter phage system hmdUra is initially incorporated into viral DNA and then part is modified into 5-(4-aminobutylaminomethyl)uracil (also called putrescine) [33,34]. Restriction enzyme digest patterns (data not shown) make it unlikely that CBA120 has a further such modification.

### Membrane Proteins and Potential Lysis Genes

Using TMHMM and Phobius, 12 predicted membrane-spanning proteins have been identified in CBA120 (indicated in green in Figure 6). Some of these proteins are highly conserved among T4-like phages, but none of their specific functions have been identified. No holin or free lysin-encoding genes have yet been detected in CBA120, though the clearing of the culture at a specific time and the apparent presence of a form of lysis inhibition strongly suggest that they exist.

### Structural Proteins: Use of ViI Proteomic Analysis

Pickard et al., 2010 [20] carried out a very detailed proteomic analysis for ViI. They identified 41 particle-associated proteins, including the products of 18 previously-uncharacterized ORFs along with all of the structural proteins predicted by genomic analysis. All of those particle-associated proteins except for the putative tail spikes and adjacent fiber have orthologs in CBA120; these latter four have clear homologs in all three phages, though with substantial differences between them. Where the specific functions are unknown for proteins whose ortholog in ViI were identified as being particle-associated, they have been indicated in turquoise in the CBA120 genome diagram (Figure 6), while the rest are shown in dark blue.

### Baseplate Lysozyme

In addition to the lysozyme responsible for ending the infection cycle, members of the T4 family generally encode a second lysozyme, usually related to the first, embedded in the central portion of key baseplate protein gp5. This aids penetration of the peptidoglycan layer during the initial infection process. The 537 AA protein identified in CBA120 as a gp5 homolog lies at genomic position 46,626-48,239. Its Vi1 ortholog was observed in Pickard's proteomic analysis [20]. It does not have the usual identifiable lysozyme, but a central region was detected between AA 150 and 282 which contains a putative CHAP domain -- a δ-D, L-glutamate-specific amidohydrolase with functions including "peptidoglycan hydrolase" [35], and belonging to the TIGRO2594 family of the NLPC_P60 superfamily. Flanking the TIGRO2594 domain are two regions, AA 3-109 and 334-437, that are also found on either side of the catalytic region in the bacteriophage T4 gp5. Also, a homolog of the N-terminal domain of the bacteriophage T4 gp5 region is found between AA 46 and 109; in T4, this domain has been identified as interacting with gp27 [25].

### Putative Tail Fibers and Spikes

Consistent with their disparate host ranges, the major differences between the genomes of CBA120, ViI and AG3 lie in the genes encoding their host-recognition elements -- CBA120 orfs 210-213 (Table 2) -- though there are clear relationships. Orfs 210, 212 and 213 encode spike proteins, as has been discussed for ViI [20]. The N-terminal 163 amino acids of the tail spikes encoded by CBA120 orfs 210 and 212 are very similar to each other and, from amino acid 23 on, to amino acids 248-490 of the orf 213 tail spike. From that point on, they diverge markedly. Much of the rest of orfs 210 and 212 show relationships to phage K1F, including its endosialidase region, and to podovirus EcoDS1 as well as to AG3 orf 207, while CBA120 orf 213 is more like AG3 orf 213 over most of its length. The conserved N-terminal segment of the three CBA120 tail spikes is also closely related (> 80%) to the N-terminal region of the published tail-spike protein of *Salmonella enterica *serovar Typhimurium phage Det7, the first myovirus demonstrated to use a podovirus-like tail spike [36]; the authors determined that this region is what binds to the tail, and determined the crystal structure of the rest of the spike, which looks much like that of phage P22. (The still-unpublished full sequence of Det7 indicates that it is also a member of this family (Sherwood Casjens, personal communication)). It is also > 60% related to the same regions of the ViI 846 aa and 596 aa tail spikes. The region from aa 103 to 211 of CBA120 orf 210 shows 45% identity with aa 156 to 264 of the tailspike gene of coliphage K1F, and similar strong identities are seen between this region of K1F and the comparable regions of the gp 212 and 213 tailspikes. The N-terminal 225-amino acid extension on the spike encoded by orf 213 appears to be unique to the ViI group of phages; one possibility is that it is involved in transferring the signal to contract from that spike to the myovirus tail structure after the spikes bind. CBA120 orf 211 encodes a putative tail fiber, with its primary relationships to similarly located orfs of ViI and AG3 throughout much of its length and also substantial similarity with orf 75 of siphovirus JK06.

**Table 2:** Properties of CBA120 Tail Spikes and Fiber

CBA120 Orf	210	212	213	211
Size (amino acids)	770	628	1037	922
Putative product	Tail spike	Tail spike	Tail spike	Fiber?
Region in CBA120	118,908- 121,20	124,151- 126,034	126,134- 129,224	121,273- 124,038
**Putative endo-N-acetylneuraminidase (phage K1F)**				
Region in CBA120	103-211	102-179	428-493	N/A
Region in phage K1F	156-264	156-235	156-223	N/A
% Identity	45	47	58	N/A
E value	2e-17	7e-12	9e-12	N-A
***N*-terminal anchor (relationship to phage Det7)**				
Region	1-163	1-348	8-230	None
% Identity	71	52	49	N/A
E value	3e-58	1-54	2e-31	N/A

Regions indicate the location of corresponding amino acid residues.

## Discussion

Our extensive molecular and physiological knowledge about the T4 and T5 phage families and the recent explorations of this new ViI group of phages are proving important in understanding phage ecology and pertinent to the construction of cocktails targeting *E. coli *O157:H7. The genomic analysis reported here clearly identifies CBA120, ViI and AG3 as very distant and unusual members of the T4 superfamily of phages. The Vi typing phages for *S. enterica *serovar Typhi were first described over 70 years ago [37] and morphologically characterized by Ackermann [19]. They were successfully used to treat typhoid fever in the 1930's and 1940's in Los Angeles and Montreal [38]. However, their physiological properties were not well explored and their genomes were only recently sequenced [20]. Phages like ViI have been reported targeting *Rhizobium meliloti *[19,39], *Acinetobacter *[40,41] and *Bordetella avium *(Ackermann, unpublished data) but have been little studied. Bacteriophage CBA120 is the first ViI-like phage identified that targets *E. coli*, and, with *Shigella *phage AG3 [22], the first of this group to be extensively explored both physiologically and genomically.

*E. coli*, a facultative aerobe, grows in the intestinal tracts of warm blooded animals, and phage CBA120, isolated from the feces of feedlot cattle using *E. coli *O157:H7 strain 12900, binds very efficiently and produces large bursts of phage in O157 both aerobically and anaerobically. Interestingly, at high multiplicity of infection the phage exhibits lysis inhibition, reported previously only in T4 and a few close relatives, with a significant delay in lysis time and a burst size of > 1000. CBA120 is highly specific for O157 and infects most O157:H7 strains. Only one other *E. coli *strain (ECOR 70) has been found that it can infect, and none of the standard test battery of *Salmonella, Shigella *and other pathogenic enteric bacteria routinely used at the USDA Agricultural Research Center in College Station, Texas were susceptible.

Relatives of *E. coli *phage T4 constitute one of the most ecologically and genomically diverse groups of phages known [28,30,42] and are major components of therapeutic cocktails used to target enteric bacteria in various parts of the world [43,44]. Sequenced members of the "T4 superfamily" now include phages of enterobacteria, *Acinetobacter, Aeromonas, Delftia, Vibrio, Prochlorococcus *and *Synechococcus*. They all share at least 33 genes that have resisted major divergence throughout a long period of evolution and have been used to develop a useful analytical tool, "core genes" [45,46]. Further groups of their genes are broadly shared within subsets of these T4-related phages, presumably reflecting adaptations to various ecosystems. All of these core genes except for gene 34, encoding the proximal arm of the tail fiber, are present in some form in CBA120, AG3 and ViI, whose structural-protein genes are often more closely related to those of the T4-like cyanobacterial phages than to similar phages from enteric sources. Nearly 70 of their genes are related to genes of *D. acidovorans *phage φW-14 [47] (GenBank accession number GQ357915.1).

The number and diversity of sequenced T4-related phages, the extent of conservation in many of the proteins involved, the depth of knowledge of the T4 tail structure, assembly and infection process [25], and the very thorough proteomic study of the ViI phage particle [20] now lay the groundwork for exploring the evolution of the complex myoviral tail structure. CBA120, AG3, ViI, φW-14 and 16 fully sequenced cyanophages all show substantial conservation of the proteins of the neck, tail tube and sheath proteins and most of the proteins found in the central part of the T4 baseplate: gp48, gp53, gp25 and gp6 [42]. The outer T-even baseplate proteins - gp7, 8, 9, and 10 -- are all missing in the ViI group. So are all parts of T4's long tail fibers for reversible adsorption, the short tail fibers involved in irreversible adsorption (gp 11 and 12), and gp29, T4's "tape-measure protein", determining the length of the tail. It seems likely that some of the new proteins detected in the proteomic studies of ViI take the place of some of these missing proteins in the baseplate structure. Likely candidates include the products of CBA120 orfs 45 and 47, which cluster with the homologues of T4 genes 54 and 48, and of orfs 209, 215 and 216, clustering with the T4 gp6 homologue and the CBA120 tail spikes.

Of particular interest is comparative analysis of the central baseplate hub protein gp5, which plays a fundamental role in tail-tube penetration of the peptidoglycan layer. A lysozyme is embedded in gp5 for both T4 and CBA120, but the CBA120 lysozyme is of apparent bacterial rather than phage origin and very different from the one involved in the T4 infection process, which is related to the lysozyme T4 uses to exit the cell. In the case of CBA120, no holin or lysis enzyme for ending the infection cycle has yet been identified. Such enzymes are highly variable [48].

### Host-Cell Recognition Elements

The presumed adhesion elements of CBA120 include spike-like proteins, related to the tail-spike proteins of ViI [20] (genes ViOI_170, 171 and 172) and to orfs 207 and 212 of AG3 [22]. The three CBA120 spike proteins (encoded by orfs210, 212 and 213) are distantly related to each other, and are very similar in their N-terminal regions (Table 2). This *N*-terminal portion is also very similar to the corresponding region of the sequenced and crystallized spike protein of *Salmonella *phage Det7 [36] and contains a segment related to the endosialidase in the spike of podovirus K1F [49]. The published crystal structure of the Det7 spike suggests that the N-terminal end is responsible for the interactions between the tail spikes and the baseplate and/or the fibrous protein shown to surround the baseplate in ViI [20], while the C-terminal and central domains form the actual adhesin. The protein encoded by CBA120 orf 213 is significantly longer than those encoded by orf 210 and orf 212 (1037 amino acids vs. 771 and 628, respectively); interestingly, orf 213's extra amino acids lie at the N-terminus of the protein, in front of the conserved baseplate-binding region. The proteomic analysis of ViI indicated that all three of the tail-spike proteins and the apparent tail fiber encoded by orf 211 are being incorporated into phage, though it was not possible to distinguish whether all are expressed in each infected cell or on each phage particle. The SP6 group of podoviruses, including Salmonella typhii typing phage ViIV, each encode two tail spikes that can target two different receptors, though only in the case of KI-5 are the two receptor targets known [50]. The modular property of the genes would be expected to facilitate recombination between tail spikes, potentially broadening the host range.

CBA120 orf 211, the fourth member of this cluster of CBA120 genes, encodes a purported tail fiber. Up to aa 254, it is virtually identical to the protein encoded by ViI_171c and also has substantial similarity to AG3 orf 210. However, beyond that point CBA120 orf 211 is very different from the other ViI-like phages. It shows high similarity over the rest of its length only to purported tail-fiber proteins of coliphages phiV10, a podoviral prophage in *E. coli *O157 (NCBI Reference Sequence: YP_512279.1) and siphovirus JK06 (isolated in Israel and reportedly highly specific for *E. coli *O157) (YP_277515). In contrast, the rest of the fiber of AG3 encoded by orf 210 is most closely related to the putative tail fiber proteins of *Erwinia amylovora *phage Era103 (YP_001039674.1) and of *Escherichia *myovirus rv5 (YP_002003543) [16]. AG3 orf 213 seems to encode a second fiber, rather than a third spike as for CBA120. It is most closely related to the tail fibers of T7-like Klebsiella phage K11 (YP_002003830) and *Prochlorococcus *T4-like myovirus P-SSM2 (YP_214526.1)

CBA120 is by far the most specific phage we have seen to date with regards to host range. It is also the first T4-related coliphage that has been reported to use tail spikes, with their concomitant enzymatic activity, as well as a tail fiber. The similarity of CBA120's tail fiber to those of two very different phages specific for O157 suggests that the fiber also plays an important role in host-cell recognition. Two other observations appear relevant in considering the interactions between CBA120 and *E. coli *O157. First, the O157 polysaccharide component is one of the few known to be produced in free form, able to produce a capsule around the cell (under separate control) that modulates its infectivity as well as being bound up in the LPS [51,52]. Secondly, the O157 LPS is one of only three of the coli O antigens reported to date that is found in some strains of *Salmonella *as well as in *E. coli *[53].

These observations support the possibility that the phage originally developed infecting *Salmonella *and migrated relatively recently to *E. coli *O157, with the tail-spike enzyme, which had presumably evolved in an O157 Salmonella strain, particularly useful in dealing with the *E. coli *O157 capsule. That, in turn, would help explain how CBA120 can be so closely related to phage ViI, with all but 17 genes in common and very similar in sequence and everything in the same order, as seen in Figure 5 - an extremely unusual degree of interspecies closeness among these larger phages.

### Base Composition

The evidence strongly suggests that, like T4, phage CBA120 uses a non-canonical base in its DNA; however, rather than substituting hmdCyt for Cyt, it appears to substitute hmdUra, or a derivative thereof, for thymidine. As shown here, CBA120 cannot incorporate ^3^H-dThd into its DNA, and its "thymidylate synthase" bears resemblance to the hmdUra synthases of φW-14, YuA and SPO1 (as do those of ViI and AG3). Also, it makes a dNMP kinase which is most similar to that for *Pseudomonas *phage YuA, which is also purported to incorporate hmdUMP into its DNA. Furthermore, the CBA120 genome is not susceptible to attack by those restriction enzymes previously shown to be inhibited by such a substitution (data not shown). It appears likely that its dUTPase/dUDPase is also a dTTPase/dTDPase (in parallel with that enzyme in T4 also being a dCTPase/dCDPase). Otherwise, some of the labeled dT should get in using the host dTMP kinase even if the hmdUMP kinase wouldn't work on dTMP coming from outside (or from host DNA breakdown).

The ViI-like family seems to be the first group of enteric phages to make use of hmdUra. It is not yet clear what advantages this brings besides protection against some restriction enzymes, but it is possible that the use of hmdUra also allows this phage family to infect a wider range of bacterial species. For example the hmdUra modification may play a significant role in allowing ViI phage to infect not only *S*. Typhi but also other species that possess the Vi capsule, such as some *S*. Dublin and *S*. Typhimurium isolates and *Citrobacter freundii *(data not shown). The presence of the new hydroxyl group in the major groove is sometimes used to enable further modifications, such as the protective glycosylation of the T-even phage DNA and the formation of putrescine in the DNA of ΦW14 that allows the packaging of substantially more DNA. The substitution of hmdCyt for Cyt in the DNA of T4 and its many closely-related phages protects them from degradation by many restriction enzymes. This substitution also allows T4 to selectively degrade the cytosine-containing DNA of its hosts and other phages, initiated by a pair of endonucleases, endo II (which nicks the DNA) and endo IV (which cleaves the single-stranded DNA opposite the nicks). Furthermore, T4 has developed a method of blocking transcription of all cytosine-containing DNA (as discussed below under gene expression). In *B. subtilis *phage SPO1, the one genetically well-characterized phage that uses hmdUra rather than dThd in its DNA, the middle-mode genes are preferentially transcribed from DNA that has hmdUra rather than dThd [32].

Whatever the nature and effect of the modification, we have determined that CBA120 is not protected from the mechanisms T5-like siphovirus CEV2 uses to take over the cell; in high-MOI coinfection, CEV2 has a normal burst size and totally suppresses CBA120 (unpublished data). In contrast, the T4-like myovirus CEV1 suppresses CEV2 while itself making a normal burst of phage [12].

### Gene Expression

The presence of homologs of T4 gp55 and gp33 in the CBA120 genome suggests that, like T4, late transcriptional control is exerted by producing a new, highly unusual sigma factor, leading to a total change in promoter recognition when it interacts with the host RNA polymerase. The T4 gp55 is much smaller than the host's σ^70 ^and interacts only with the -10 region of the promoter, not with a -35 region. Instead, it interacts directly with the host RNA polymerase and the DNA replicative complex (through the mediation of gp33 and gp45), which acts as a "moving enhancer", in the words of P. Geiduschek, whose laboratory did much of the work in this area [29]. This effectively links capsid protein production and DNA synthesis.

The situation with regard to early and middle gene recognition is much more complex. Neither the ViI-like phages nor the cyanophages have homologs of gpalt or gpmodA, which, in T4 and many related phages, ADP ribosylate one (Alt) and later both (ModA) of the alpha subunits of the host RNA polymerase, modulating early transcription. They also do not have any MotA, AsiA or other obvious mechanism of regulating the slightly later transcription of the genes involved in nucleotide biosynthesis and DNA replication. Identifying the control signals and proteins involved in each case can be expected to require extensive experimental work, as it did in T4 [54,55]. In particular, points where the direction of transcription diverges must utilize a still-unidentified promoter in each direction.

CBA120 has a few canonical *E. coli *σ^70 ^promoters, as also seen in T4. However, their role in phage gene regulation is not clear. T4 rapidly redirects most host RNA polymerase from about 650 σ^70^-dependent to 39 T4-specific immediate early promoters. This rapidly turns off host transcription even in T4 mutants lacking the Alc protein, which blocks the elongation of transcription on cytosine-containing DNA [56,57]. T4 also carries in its capsid a protein, gp*alt*, which adds an ADP ribose to one of the two alpha subunits of the RNA polymerase and may facilitate this transition, but little difference is seen in its absence. We have been unable to identify potential early promoters in CBA120 on the basis of informatics, and as detailed above it has no apparent homologues of the proteins involved in T4 early and middle-mode gene regulation. A similar lack of apparent separate middle-mode transcription signals has been reported for other distantly T4-related phages, including vibriophage KVP40 and the cyanophages; it has been suggested that distance from the promoter and anti-termination may be more involved in regulation of delayed-early genes there [30].

### Lysis and Lysis Inhibition

As mentioned above, neither the lytic enzyme nor a holin to let it reach the peptidogycan layer and thus control lysis timing has of yet been identified for CBA120, or for ViI or AG3. This is of special interest in trying to understand the apparent lysis inhibition observed above in high-MOI infections. For T4, lysis inhibition has been shown to depend on an interaction between the holin and a labile periplasmic antiholin, encoded by the *rI *gene, which is somehow able to detect superinfection at any point until just before the normal time of lysis and delay lysis for several hours [58-60]. It will be very interesting to see whether the lysis-inhibition mechanism is related in T4 and CBA120, even though the proteins involved seem to be very different.

## Conclusion

CBA120 is representative of a newly described group of phages that appear to be of interest evolutionarily, ecologically and historically. Recently, such phages, related to *Salmonella *Typhi phage ViI, have been discovered infecting a variety of different *Enterobacteriaceae*. The major distinguishing feature between them and other T4-like phages seems to be their strategy in host recognition, with the ViI-like phages employing spikes related to ones commonly seen in podoviruses, incorporating an endoglycosidase into their structure, rather than relying on the long tail fibers seen in the other T4-related phages. On the other had, the reticular network that sometimes appears around the baseplate (Figure 1, arrow B), also seen in ViI, has not been reported in podoviruses. While many tail elements are well conserved in these and other T4-related phages, the outer baseplate proteins are very different, with no apparent homologues of the T4 gp12, responsible for the irreversible final step in host binding and different also from those of cyanophages. Each of the phages that have been sequenced to date in this ViI group encodes three different but related spikes, one of which contains a 225-amino acid N-terminal extension that could possibly transmit information on binding that instigates tail contraction. It is not yet clear just what role these play in determining their host ranges, or even whether each given phage carries all three different spikes.

Another major apparent difference in infection strategy between the three known major groups of T4-related phages, carrying the core T4 set of structural and replication-related genes, lies in their choice of pyrimidine bases. While the cyanophages, other oceanic phages and a few of the phages infecting enteric bacteria use the canonical bases cytosine and thymine, it appears that those ViI-like phages sequenced to date produce and incorporate hmdUra rather than thymidine, just as most of the previously-known T4-like phages infecting enteric bacteria use hmdCyt rather than cytosine in synthesizing their DNA. It will be very interesting to compare the consequences of these major strategic choice differences between T4, the T4-like cyanophages and these ViI-like phages which all share a substantial core set of the genes encoding most morphogenetic and replicative proteins.

## Materials and methods

### Bacteria and Bacteriophages

Phage CBA120 and CBA6 were isolated from a feedlot in the southern plains region of the U.S.A., as previously described [17,18]. Phages CEV1 and CEV2 were isolated from two different flocks of Texas sheep [12,13]. All three phage were propagated on *E. coli *O157:H7 NCTC 12900, which lacks the Shiga-toxin (Stx) genes (Biosafety level 1; obtained from the American Type Culture Collection: ATCC 700728). Phage stocks were prepared as per our standard lab method where the host organism, *E. coli *O157:H7 NCTC 12900, was grown in tryptic soy broth (Bacto-TSB, Becton Dickinson Cat.# 214530) to an OD_600 nm _~0.2 before adding phage to an MOI ~0.01-0.1. The mixtures were then returned to a shaking water bath (180 rpm) at 37°C. After 90 min a few drops of chloroform (CHCl_3_) were added to complete lysis of the infected cells and the stock was placed in the dark at room temperature for ~12 h allow any free DNA to be degraded. The phage culture was centrifuged for 20 min at 5500 × *g *to remove bacterial debris, followed by 2 h at 15,000 × *g *to precipitate the phage. The phage pellets were allowed to slowly resuspend for 24-48 h at 4°C into phage buffer (1 mM Tris, pH 7.6, 0.1 mg mL^-1 ^gelatin, 4 mg mL^-1^, NaCl), yielding a final titer > 10^11 ^PFU mL^-1^, and stored at 4°C.

### Host Range Determination and Efficiency of Plating (EOP)

The host ranges and efficiencies of plating were determined as described [61]. Square plastic plates embossed with a 6 × 6 grid containing TSA (1% w/v agar) were overlaid with 4 mL molten top TSA (0.3% w/v agar) containing 0.3 mL of an exponential culture of the host strain to be tested. Once dry, 10 μL of each phage stock (~10^8 ^PFU mL^-1 ^on strain 12900) was spotted on the plate and incubated at 37°C overnight. For those testing positive, a series of dilutions was then spotted in similar fashion to determine the EOP (phage titer on the strain being examined/phage titer on NCTC 12900, for CBA120). Bacterial strains tested included standard lab strains; the 72-member *E. coli *collection of reference (ECOR) from the University of Rochester, NY [62]; a set of 15 pathogenic *E. coli *O157:H7 strains from the Federal Disease Investigation Unit (FDIU-Washington State University); and a group of 107 *E. coli *strains from cows with post-partum metritis isolated at The Evergreen State College by Mike Paros and Tom Denes and at Cornell University, Ithaca, New York by Rodrigo Bicalho.

### Transmission Electron Microscopy

Phages for electron microscopy were sedimented for 60 min at 25,000 × *g *using a JA-18.1 fixed angle rotor. This was followed by two washes in 0.1 M neutral ammonium acetate under the same conditions. Purified phages were deposited on carbon-coated copper grids, stained with 2% (w/v) potassium phosphotungstate (pH 7.0) and examined in a Philips EM 300 electron microscope operated at 60 kV. Magnification was monitored with T4 phage tails.

### High MOI Aerobic and Anaerobic Infections

Both aerobic and anaerobic infections of CBA120 were carried out in TSB, as previously described [12,13]. Aerobic experiments used shake flasks at 37°C with agitation (180 rpm). Anaerobic experiments were conducted in anoxic TSB in butyl-rubber-sealed serum bottles under an N_2 _head space at 37°C with agitation (180 rpm). All transfers were carried out aseptically using N_2 _flushed syringes. Once the culture had reached OD_600 nm _~0.3 (mid-exponential phase), phage were added at an MOI of ~5 (in ~1:20 of the culture volume) with rapid mixing. Samples were periodically taken until lysis to determine cell density (OD_600 nm_), enumerate total phage (after immediate treatment with 100 μL CHCl_3_, PFU mL^-1^), infective centers (PFU mL^-1^) and bacterial survivors (CFU mL^-1^). Phage samples were titered in duplicate using our standard double layer technique aerobically on a lawn of strain 12900. Bacterial survivors were similarly titered on TSA plates.

### Single-Step Growth Curve

This method of precisely determining the eclipse time (until first appearance of mature phage) and latent period (until the start of lysis) was carried out as described by Carlson (2005) [63]. Infection was carried out aerobically in TSB, as described above, at an MOI of 0.1. Five min after infection, the culture was diluted in TSB and 10^2^, 10^4^,10^5 ^and 10^6 ^fold dilutions were further incubated with agitation at 37°C. Samples for infective centers were plated directly out of flasks at the appropriate dilution, while samples for total phage were taken from an appropriate flask into tubes with chloroform and allowed to sit for at least 15 min to let the cells lyse before further dilution and plating.

### ^3^H-dThd DNA Labeling

A 2 mL sample of culture was transferred 5 min after phage infection to a 10 mL flask containing 0.1 mL dAdo (2 mg mL^-1 ^in H_2_O) and 0.1 mL dThd stock (100 μg mL^-1 ^dThd, 10 μCi mL^-1 ^methyl ^3^H-dThd) and kept shaking at 37°C. dAdo is added to allow the dThd to be taken up by *E. coli *without being degraded by phosphorylase and hydrolases, as described by Boyce and Setlow, 1962 [21]. For each sample, 50 μL was transferred to a 1 cm square of 3 MM filter paper. After 1 min, the disk was dropped into a beaker of ice cold 10% (v/v) acetic acid (about 5 mL/disk). At least 15 min after the final sampling, the acetic acid was poured off and the disks washed twice for 15 min with the same volume of 5% (v/v) cold acetic acid. A final 15 min rinse in 5 mL/disk of ice cold 100% ethanol was used to wash out the acid and prevent undue quenching. The disks were dried overnight and counted in a Packard Tri-carb 2200CA liquid scintillation counter, using 10 mL of Perkin-Elmer CyberGold (Catalogue # 6013320) as the counting fluid. A "total" sample, dried without washing, was taken from each flask at the start and end of the labeling experiment to confirm the amount of label added.

### DNA Isolation for Sequencing

A 1.5 mL aliquot of CBA120 (> 10^12 ^PFU mL^-1^) in phage buffer was added to phenol (700 μL) in an Eppendorf tube and microcentrifuged at 13,5000 rpm for 10 min. The supernatant was subjected to a second identical phenol treatment, followed by two similar extractions with chloroform (700 μL). The DNA was precipitated with 1.5 × vol 0.3 M sodium acetate-buffered cold ethanol (pH 5.3), pelleted by microcentrifugation at 13,500 rpm for 45 min, and washed in 70% (v/v) ice-cold ethanol. The pellet was resuspended in TE buffer (10 mM Tris-HCl, 1 mM EDTA, pH 7.5). An identical procedure was used to extract DNA for restriction enzyme digests. The DNA was subjected to pyrosequencing (454 technology) at the McGill University and Genome Québec Innovation Centre (Montreal, QC, Canada).

### Genome Annotation

CBA120 was annotated with the aid of a variety of online tools (http://molbiol-tools.ca; NCBI) and ARTEMIS [64]. The genome was initially subjected to automated annotation using AutoFACT [65] following which all open reading frames (ORFs) were confirmed using Kodon total genome and sequence analysis software, version 2.0 (Applied Maths Inc., Austin, TX. USA). Annotation was then manually curated as described previously [64] using Artemis software [66] to collect the data and facilitate annotation. The predicted proteins were compared again the PFAM database of protein domain hidden Markov models [67] and investigated for helix-turn-helix structural motifs. The results of all the analysis were assembled using Artemis. Genes were identified from among the predicted coding sequences (CDSs) based on the presence of ATG, GTG, CTG or TTG start codons, at least 30 additional codons, and an upstream sequence resembling the Shine-Dalgarno ribosome-binding site, GGAGGT [68], making corrections for initially miss-identified start codons. BLASTP was used to determine similarities in the global database at http://www.ncbi.nlm.nih.gov. Genomic comparisons at the proteomic level were made using CoreGenes [69,70]. Transmembrane domains were predicted using TMHMM v2.0 and Phobius [71]. Phage-encoded tRNA genes were identified with Aragorn, using the default parameters [72]. A few promoters were identified based on sequence homology to the consensus *E. coli *promoter, TTGACA (N_15-18_) TATAAT. The annotated genome sequence was submitted to the NCBI nucleotide database under accession number JN593240. Genomic comparisons were carried out using BLASTN and BLASTX [73]. Artemis Comparison Tool (ACT) was used to visualize pairwise genomic comparisons [23]. The following genome sequences were used in the genomic comparisons with CBA120 phage: phage ViI (accession number: FQ312032) and AG3 (accession number: FJ373894). Orthologs in the CBA120, ViI and AG3 phages were identified using an all-against-all reciprocal FASTA comparison of translated DNA with at least 40% identity over 80% of the length [74].

### Tree Construction

Proteins were aligned using the local pair algorithm in MAFFT with BLOSUM 30 used as the scoring matrix. The maximum likelihood topology, branch lengths and bootstraps were obtained with PhyML, where the WAG+I+GAMMA (8) model of evolution was employed. The NJ bootstraps where calculated using the Poisson distance between sequences over 100 pseudoreplicates. Parsimony bootstraps correspond to the bipartitions pattern obtained after 100 pseudoreplicates. In all cases gaps were treated as unknown states.

## Abbreviations

^3^H-dThd: Tritiated thymidine; ACT: **A**rtemis **C**omparison **T**ool; BLAST: **B**asic **L**ocal **A**lignment **S**earch **T**ool; CFU: Colony Forming Unit, a measure of the number of viable cells; Cyt: Cytosine; dAdo: deoxyadenosine; dThd: deoxythymidine; ECOR: *Escherichia coli *Collection of Reference; EOP: Efficiency of Plating: ratio of phage titer to that on *E. coli *O157:H7 12900; GP: Gene product; HMase: Hydroxymethyltransferase; hmdCyt: 5-(hydroxymethyl)deoxycytosine; hmdUra: 5-(hydroxymethyl)deoxyuracil; MAFFT: **M**ultiple **A**lignment using **F**ast **F**ourier **T**ransform; MOI: Multiplicity of Infection, ratio of infective phage particles to vulnerable hosts; N/A: not applicable; PhyML: **Phy**logenetic estimation using **M**aximum **L**ikelihood; PFU: Plaque Forming Unit, a measure of the number of viable viral particles; TMHMM: **T**rans**M**embrane prediction using **H**idden **M**arkov **M**odels; TS: Thymidylate synthase; TSB: Tryptic Soy Broth

## Competing interests

The authors declare that they have no competing interests.

## Authors' contributions

EMK and ADB designed and guided the project; EMK, KSK, BB, HWA and ADB did most of the writing of the paper; HWA performed the electron microscopy; EMK, KSK, BB, AES, AC, DB carried out the physiological experiments and determined the host ranges on lab strains and the ECOR collection; TC was involved in isolation of the samples from feed lots and in their testing against a range of pathogenic enterobacterial strains, including the O157 strains; AV did the sequencing and the first round of annotation; AMK arranged for the sequencing and did much of the early annotation; EMK, KSK, BB, HA, ALT, DP carried out most of the in-depth exploration of the genome and its relationship to T4, to ViI and to AG3, and to other members of the T4-like phage family. All authors read and approved the final manuscript.
